# Genetic diversity analyses of *Lasiodiplodia theobromae* on *Morus alba* and *Agave sisalana* based on RAPD and ISSR molecular markers

**DOI:** 10.1080/21501203.2016.1232762

**Published:** 2016-10-11

**Authors:** Hong-hui Xie, Ji-guang Wei, Rong-shao Huang, X.B. Yang

**Affiliations:** a College of Agriculture, Guangxi University, Nanning, China; b Guangxi Subtropical Crops Research Institute, Nanning, China; c Department of Plant Pathology and Microbiology, Iowa State University, Ames, Iowa, USA

**Keywords:** *Lasiodiplodia theobromae*, genetic diversity, genetic variation, RAPD, ISSR

## Abstract

Genetic diversity of 23 *Lasiodiplodia theobromae* isolates on *Morus alba* and 6 isolates on *Agave sisalana* in Guangxi province, China, was studied by using random amplified polymorphic DNA and inter-simple sequence repeat molecular markers. Results of two molecular markers showed that the average percentage of polymorphic loci of all isolates was more than 93%. Both dendrograms of two molecular markers showed obvious relationship between groups and the geographical locations where those strains were collected, among which, the 23 isolates on *M. alba* were divided into 4 populations and the 6 isolates on *A. sisalana* were separated as a independent population. The average genetic identity and genetic distance of 5 populations were 0.7215, 0.3284 and 0.7915, 0.2347, respectively, which indicated that the genetic identity was high and the genetic distance was short in the 5 populations. Average value of the gene diversity index (*H*) and the Shannon’s information index (*I*) of 29 isolates were significantly higher than 5 populations which showed that genetic diversity of those isolates was richer than the populations and the degree of genetic differentiation of the isolates was higher. The *Gst* and *Nm* of 29 isolates were 0.4411, 0.6335 and 0.4756, 0.5513, respectively, which showed that the genetic diversity was rich in those isolates.

## Introduction

The fungus *Lasiodiplodia theobromae* (Pat.) Griffon & Maubl., anamorph of *Botryosphaeria rhodina* (Berk. & M.A. Curtis) Arx, is pleomorphic, plurivorous and ubiquitous soilborne pathogen in the tropics and subtropics (Úrbez-Torres et al. 2008). As a plant pathogen, *L. theobromae* associated with up to 500 plant hosts and caused numerous diseases, including gummosis, leaf necrosis, dieback, collar rot, root rot, fruit rot, leaf spot, decline, leaf blight, cankers and witches’ broom and so on (Úrbez-Torres et al. 2008). It also occurs as an endophyte (Mohali et al. 2005; Rubini et al. 2005). Mulberry root rot caused by *L. theobromae* in Heng County of Guangxi Province, China, was first reported in 2014 (Xie et al. 2014) and diseased area was about 2400 ha, where approximately 300 ha mulberry was seriously diseased with the diseased and mortality rate more than 80%. It was found that this disease also occurred in many other counties of Guangxi Province where mulberry was cultivated.

Colonies of *L. theobromae* were initially white, with fluffy and aerial mycelia, and overgrew the Petri dish in 4 days. Then, the colonies turn grew to black and spreading with superficial dark-branched septate mycelia. Black colour of mycelia on reverse sides of Petri plates was visible. The conidia of the pathogen were initially unicellular, hyaline ellipsoid to subovoid. Mature conidia were dark brown, bicelled, thick walled and ellipsoid.

The molecular markers such as random amplified polymorphic DNA (RAPD) and inter-simple sequence repeat (ISSR) have been extensively used to investigate the genetic diversity of plant pathogens at strain and population level (Wu et al. 2000; Liu et al. 2014; Chen et al. 2015). Genetic cluster dendrogram based on the results of molecular markers often keeps a relationship with the geographical region, host, pathogenicity, vegetative compatibility group and other aspects of the tested isolates, and the genetic diversity and difference of intra- and inter-species from different sources could be understood by analysing the dendrogram (Shafagh et al. 2008; Baysal et al. 2010). Dendrogram generated based on RAPD and ISSR divided the 158 isolates of *Botryosphaeria* into three groups and those groups were identical with the morphological characteristics and cultural properties of those isolates (Zhao 2007). The aim of this study was to evaluate genetic diversity of some *L. theobromae* isolates collected from *Morus alba* and *Agave sisalana* by RAPD and ISSR molecular markers and reveal the genetic variation and the correlation between genetic diversity and geographical locations.

## Materials and methods

### Isolates and identification

**Figure 1.**
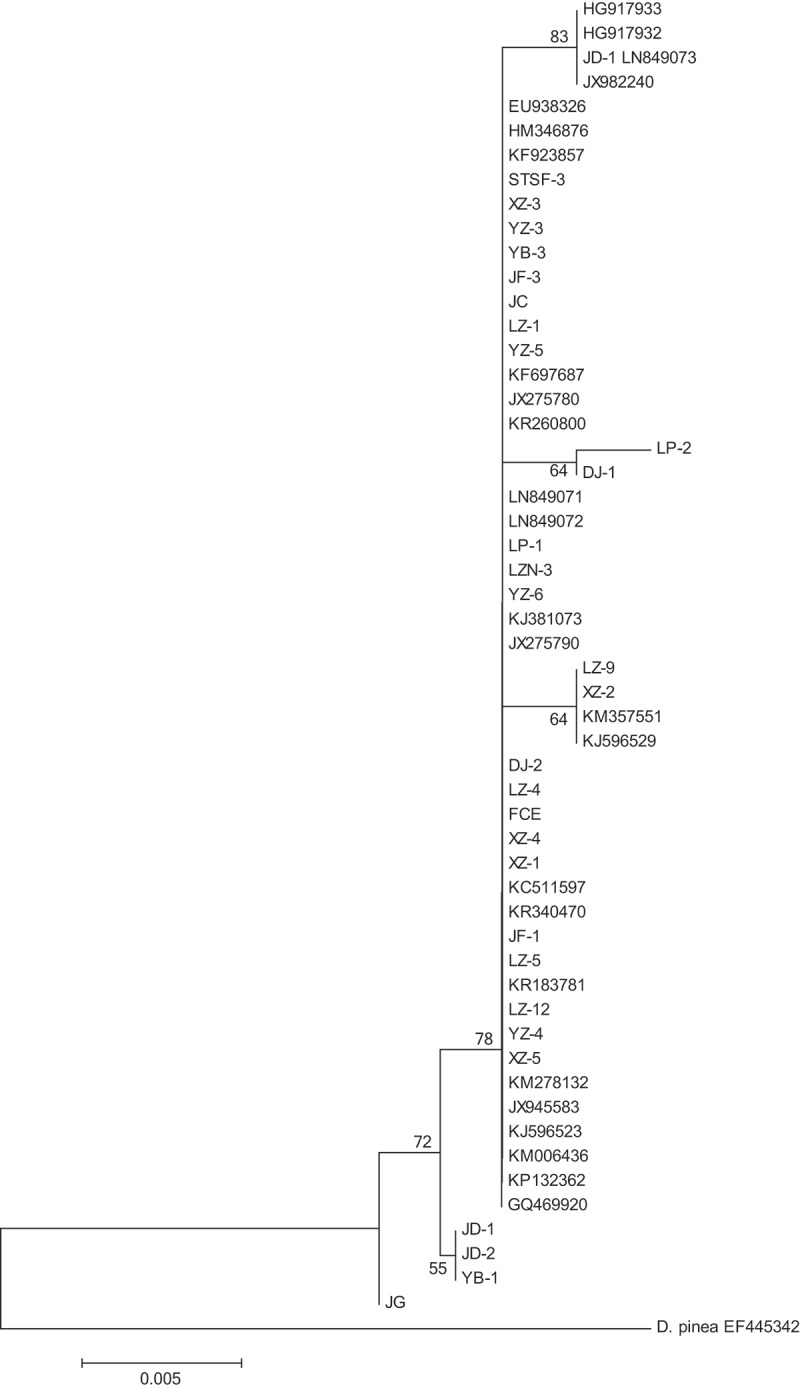
Phylogenetic tree of the tested isolates.

Twenty-three isolates and six isolates of *L. theobromae*, respectively, on *M. alba* and *A. sisalana* Perr. ex Engelm. collected from geographical locations were used for analysing the genetic diversity (Table 1), which were confirmed to be the pathogen of mulberry root rot and sisal leaf spot disease on *A. sisalana* according to the Koch’s postulate, respectively, and were identified as *L. theobromae* based on morphological characteristics and sequences of the rDNA internal transcribed spaces (rDNA-ITS) and nuclear translation elongation factor l-α (EF1-α) previously. Phylogenetic tree based on the rDNA-ITS sequences of the 29 isolates was made (Figure 1).

**Table 1.** Isolates for genetic diversity analysis.

Number	Isolates code	Host	Geographical location	BLAST search results or GenBank accession number
1	DJ-2	*Morus alba* L.	Heng County, Guangxi	99% with KM278132
2	DJ-1	*Morus alba* L.	Heng County, Guangxi	100% with KF697687
3	FCE	*Morus alba* L.	Heng County, Guangxi	99% with KJ381073
4	STSF-3	*Morus alba* L.	Heng County, Guangxi	99% with KF923857
5	YB-1	*Morus alba* L.	Heng County, Guangxi	HG917932
6	YB-3	*Morus alba* L.	Heng County, Guangxi	99% with JX275780
7	LP-1	*Morus alba* L.	Heng County, Guangxi	99% with JX275790
8	LP-2	*Morus alba* L.	Heng County, Guangxi	99% with JX945583
9	LZ-5	*Morus alba* L.	Luzhai County, Guangxi	99% with KM357551
10	LZ-9	*Morus alba* L.	Luzhai County, Guangxi	99% with KR260800
11	LZN-3	*Morus alba* L.	Luzhai County, Guangxi	99% with JX982240
12	LZ-4	*Morus alba* L.	Luzhai County, Guangxi	99% with HM346876
13	LZ-1	*Morus alba* L.	Luzhai County, Guangxi	100% with KC511597
14	LZ-12	*Morus alba* L.	Luzhai County, Guangxi	99% with KR183781
15	YZ-3	*Morus alba* L.	Yizhou County, Guangxi	99% with KJ596529
16	YZ-4	*Morus alba* L.	Yizhou County, Guangxi	99% with KR340470
17	YZ-5	*Morus alba* L.	Yizhou County, Guangxi	99% with HG917933
18	YZ-6	*Morus alba* L.	Yizhou County, Guangxi	100% with KR340470
19	XZ-1	*Morus alba* L.	Xiangzhou County, Guangxi	99% with KJ596523
20	XZ-2	*Morus alba* L.	Xiangzhou County, Guangxi	99% withEU938326
21	XZ-3	*Morus alba* L.	Xiangzhou County, Guangxi	99% with KM006436
22	XZ-4	*Morus alba* L.	Xiangzhou County, Guangxi	99% with KP132362
23	XZ-5	*Morus alba* L.	Xiangzhou County, Guangxi	99% with GQ469920
24	JD-1	*Agave sisalana* Perr. ex Engelm.	Wuming County, Guangxi	LN849073
25	JD-2	*Agave sisalana* Perr. ex Engelm.	Wuming County, Guangxi	LN849072
26	JG	*Agave sisalana* Perr. ex Engelm.	Wuming County, Guangxi	LN849071
27	JF-1	*Agave sisalana* Perr. ex Engelm.	Wuming County, Guangxi	99% with LN849071
28	JF-3	*Agave sisalana* Perr. ex Engelm.	Wuming County, Guangxi	99% with KR260794
29	JC	*Agave sisalana* Perr. ex Engelm.	Wuming County, Guangxi	99% with KR001854

### DNA extraction

Isolates were cultured on potato dextrose agar for f5 days at 28°C and the genomic DNA was extracted by using the E.Z.N.A.® Fungal DNA Min Kit (produced by OMEGA Co. Ltd.). DNA concentration was quantified on a spectrophotometer (Eppendorf Biophotometer, Hamburg, Germany) and was diluted according to experimental needs.

### Primers selection

RAPD primers with more bands and better repeatability were screened out by using fifty 10-base oligonucleotide random primers to amplify the DNA of 10 isolates. Then, the selected primers were used to amplify the DNA of the 29 isolates. For tested ISSR primers screening, 60 primers published by University of British Columbia, Vancouver, Canada, were used to amplify the DNA of 10 isolates and the selected primers were used to amplified the DNA of all the isolates. All the primers were synthesized by Sangon Biotech, Co., Ltd. Shanghai, China.

### RAPD PCR amplification

PCR amplification was carried out in a 25-µl reaction volume containing 2-µl template DNA (approximately 30 ng/µl), 12.5 µl DreamTaq™ Green PCR MasterMix (produced by Sangon Biotech, comprising dATP, dCTP, dTTP, dGTP, 0.4 mM each; 4 mM MgCl_2_; blue dye and yellow dye), 2 µl primer (10 μmol/l) and 8.5 µl ddH_2_O. The amplification was performed in a Thermocycler (My Cycler™ Thermal Cycler System 170-9703, Bio-Rad) with initial denaturation at 95°C for 5 min, followed by 45 cycles at denatured temperature 95°C for 30 s; annealed temperature was 39°C for 1 min, extended temperature 72°C for 2 min and the final extension at 72 for 15 min.

### ISSR PCR amplification

PCR amplification was carried out in a 25-µl reaction volume containing 1-µl template DNA (approximately 30 ng/µl), 1 µl primer (10 μmol/l), 0.5 µl dNTP (10 mM /μL), 2.5 µl Taq Buffer, 2 µl 25 mM MgCl_2_, 0.2 µl Taq polymerase and 17.8 µl ddH_2_O. The amplification was performed in a Thermocycler with initial denaturation at 95°C for 5 min, followed by 40 cycles at denatured temperature 95°C for 30 s; annealed temperature was subject to primer from 42 to 48°C for 45 s, extended temperature 72 for 2 min and the final extension at 72 for 15 min.

### PCR products measured

The amplified products of RAPD and ISSR PCR were size separated in 1.8% agarose gel (containing 4S Green Plus Nucleic Stain, Sangon) under 1 × TAE buffer (40 mM Tris, 20 mM acetic acid and 1 mM EDTA) at 80 V for 120 min and 100 V for 180–200 min at room temperature, respectively. All the gels were visualized under UV light and photographed using a gel documentation system (UVItec®). The sizes of amplified DNA fragments were estimated by comparison with 10 kb DNA ladder markers (BBI).

## Data analysis

Those clear, intense and reproducible amplified fragments of RAPD and ISSR were converted and normalised into a data pattern as binary matrix with “1” for presence and “0” for absence. Polymorphic regions were scored to determine the similarity among the isolates. Hierarchical clusters were analysed to construct dendrograms, using Dice’s coefficient and unweighted pair group method with arithmetic mean (UPGMA) methods with the NTSYS-PC software (Version 2.10e) (Rohlf 1998). The dendrogram was reconstructed 1000 times by repeated sampling with replacement and the frequency on which clusters formed indicating the strength of the clusters. The software POPGENE (Version 1.32) was used for statistical analysis of standard population genetics (Yeh et al. 1999). The mean gene diversity index (*H*) was calculated as *H* = (1 − ∑P*_i_*^2^), of which P*_i_* was the frequency of allele at the locus (Nei 1973). Heterozygosity and percent polymorphic loci were estimated for all populations. Genotypic diversity was calculated by Shannon’s information index (*I*). Differentiation among populations was estimated by indirect estimation of gene flow using *Gst* with *Nm *= 0.5(1 − *Gst*)/*Gst* (McDermott and McDonald 1993).

## Results

### The primers selection and amplification

**Figure 2.**
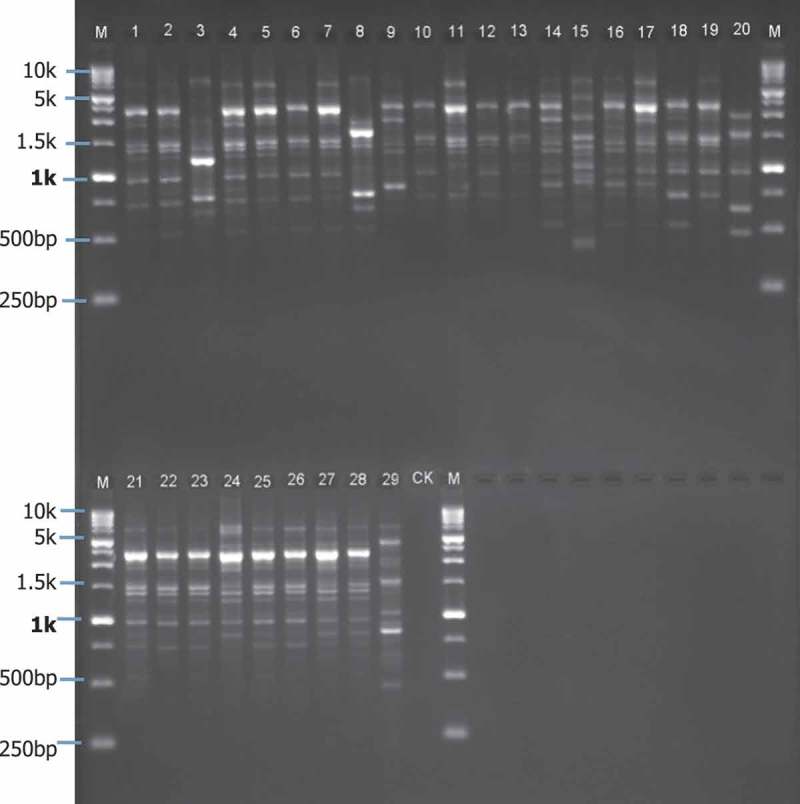
Electrophoresis of RAPD PCR products of 29 *L. theobromae* isolates obtained by primer OPC08. Lanes 1–6: Isolates from Luzhai County; lanes 7–11: isolates from Xiangzhou; lanes 12–14 and 18: isolates frome Yizhou; lanes 15–17 and 19–23: isolates from Heng; lanes 24–29: isolates from *Agave sisalana*; CK: negative control; M: marker.

**Figure 3.**
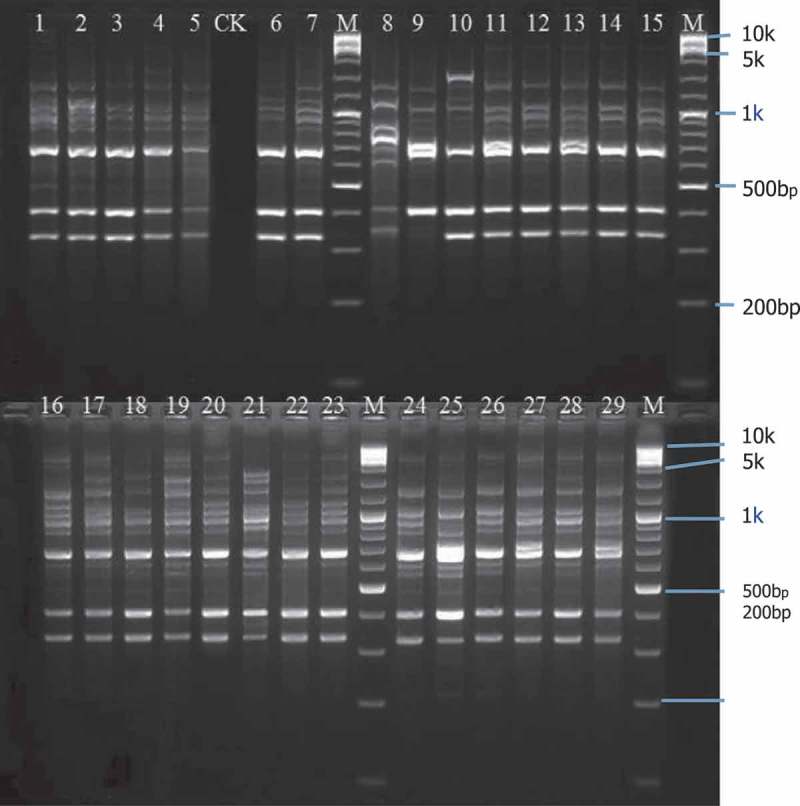
Electrophoresis of ISSR PCR products of 29 *L. theobromae* isolates obtained by primer 885. Lanes 1–5: Isolates from Xiangzhou; lanes 6–13: isolates from Heng; lanes 14–17: isolates from Yizhou; lanes 18–23: isolates from *Agave sisalana*; lanes 24–29: isolates from Luzhai; CK: negative control; M: marker.

Sixteen RAPD primers and 13 ISSR primers with more bands and better repeatability were screened out, respectively (Table 2). A total of 168 constantly amplified DNA bands between 450 and 7200 bp in length (500–4000 bp in most instances) were generated from 16 RAPD primers, among which 97.6% were polymorphic. The number of DNA bands amplified by primer OPC08 was the most and totally 13 bands were polymorphic (Figure 2). Major size of the 135 DNA bands generated with the 13 ISSR primers was from 350 to 3000 bp, and the average percentage of the polymorphic loci was 93.96%. Among those primers, 13 bands were amplified with the primer 885 and all of them were polymorphic (Figure 3).

**Table 2.** Primers selected for RAPD and ISSR.

RAPD primers				ISSR primers*			
Primer code	Sequence (5′→3′)	Annealed temperature (°C)	Amplified /Polymorphic stripes	Primer code	Sequence (5′→3′)	Annealed temperature (°C)	Amplified /Polymorphic stripes
OPA02	TGCCGAGCTG	39	11/11	810	(GA)8T	42	10/10
OPA03	AGTCAGCCAC	39	10/10	818	(CA)8G	45	12/11
OPB07	GGTGACGCAG	39	11/11	840	(GA)8YT	42	12/12
OPC05	GATGACCGCC	39	9/8	858	(TG)8RG	42	9/8
OPC08	TGGACCGGTG	39	13/13	868	(GAA)5	42	10/8
OPC11	AAAGCTGCGG	39	9/9	878	(GGTA)4	45	11/10
R08	CCCGTTGCCT	39	12/11	880	(GGAGA)3	42	9/9
R09	TGAGCACGAG	39	8/8	884	HBH(AG)7	42	10/9
R02	CACAGCTGCC	39	10/10	885	BHB(GA)7	42	13/13
R15	GGACAACGAG	39	11/11	888	BDB(CA)7	48	10/9
K20	ACGGCAAGGA	39	9/8	890	VHV(GT)7	45	10/10
S110	CCTACGTCAG	39	12/12	895	AGAGTTGGTACGTCTTGAT	45	9/9
S96	AGCGTCCTCC	39	9/9	899	CATGGTGTTGGTCATTGTTCCA	48	10/9
S56	AGGGCGTAAG	39	11/11				
S30	GTGATCGCAG	39	13/12				
S2	TGATCCCTGG	39	10/10				
Total			168/164				135/127

*B = (C,G,T); D = (A, G, T); H = (A,C,T); V = (A,C,G); R = (A,G); Y = (C,T).

### Cluster analysing of RAPD and ISSR

**Figure 4.**
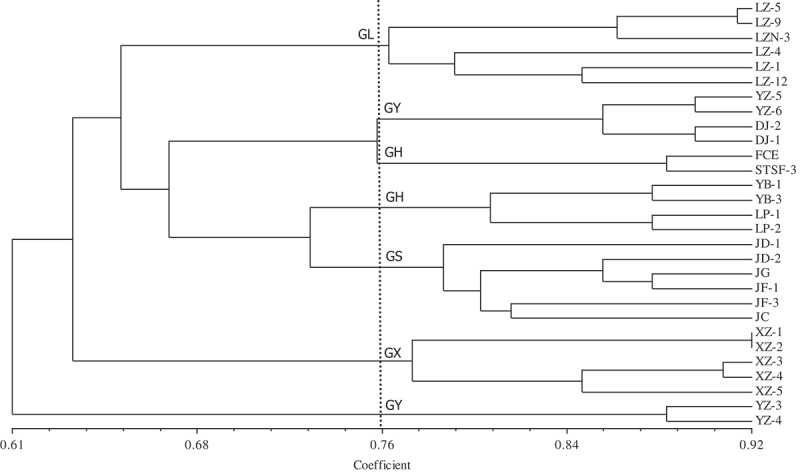
UPGMA dendrogram of 29 *L. theobromae* isolates based on RAPD analysis.

**Figure 5.**
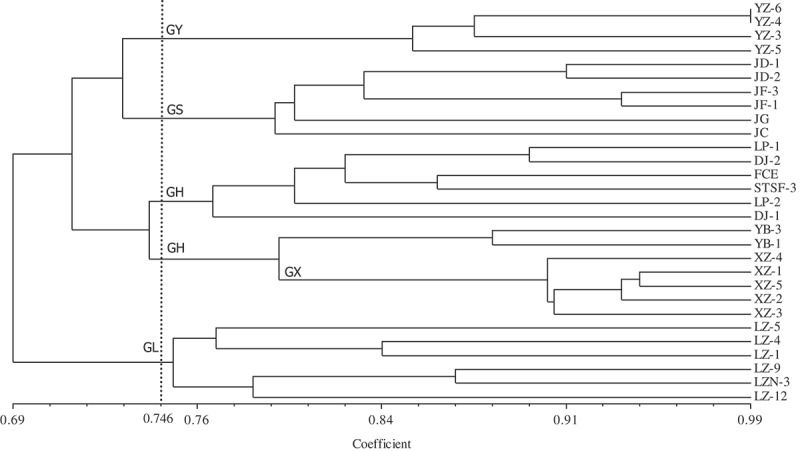
UPGMA dendrogram of 29 *L. theobromae* isolates based on ISSR analysis.

Dendrograms produced from UPGMA analysis based on Nei’s similarity coefficient of RAPD and ISSR molecular markers showed the similarity coefficient ranged from 0.61 to 0.92 and from 0.69 to 0.99, respectively, and divided the 29 isolates into 7 branches (when the similarity coefficient reached 0.76) and 5 branches (when the similarity coefficient reached 0.746), respectively (Figure 4; Figure 5). The UPGMA dendrogram based on RAPD showed that except the isolates from Luzhai (independently clustered as GL), *A. sisalana* (independently clustered as GS) and Xiangzhou (independently clustered as GX) isolates from Heng (GH) and Yizhou (GY) were clustered into two or three different groups.

The UPGMA dendrogram based on ISSR showed that the isolates from Yizhou, Luzhai, *A. sisalanal* and Heng (six isolates) were independently clustered in a branch, but two isolates from Heng and five isolates from Xiangzhou were clustered together. The dendrograms based on RAPD and ISSR indicated that most isolates clustered groups reflecting relationship with the geographical locations where those isolates were collected.

Genetic diversity of 29 isolates of *L. theobromae* collected from different hosts and geographical locations showed that those isolates were highly polymorphism. The genetic similarity coefficients and its amplitudes generated by RAPD and ISSR indicated that two molecular markers could reveal the genetic background and the degree of genetic variation among those isolates was very high.

Clusters on dendrogram were first separated all isolates as two parts, a part belonging to isolates on *M. alba* and another part belonging to isolates on *A. sisalana*. Twenty-three isolates on *M. alba* were divided into four populations, namely, Xiangzhou (GX), Yizhou (GY), Luzhai (GL) and Heng (GH) just corresponding with geographical locations where these isolates were collected.

### Genetic variation analysis

The average genetic identity and average genetic distance of the 5 populations based on RAPD and ISSR molecular markers were 0.7215, 0.3284 and 0.7915, 0.2347, respectively, which showed that there were high genetic identity and short genetic distance among those populations. The average gene diversity index (*H*) and the average Shannon’s information index (*I*) of the 29 isolates were significant higher than the 5 populations based on the RAPD and ISSR molecular markers, which indicated that the genetic diversity and genetic differentiation of isolates were higher than that among the populations.

Of RAPD molecular markers, Nei’s genetic identity and genetic distance were different among the five populations (Table 3, showed by upright numbers). The genetic identity ranged from 0.6546 to 0.7943, and the genetic distance ranged from 0.2303 to 0.4237. The maximum genetic identity appeared between the population GS and GH, and the maximum genetic distance appeared between the populations GL and GX. Parameters of genetic diversity of the five populations were calculated by PopGene software and results showed that average value of observed alleles (*Na*) of the 5 populations was 1.5476, the average value of effective alleles (*Ne*) was 1.4009, the average gene diversity index (*H*) was 0.2224 and the average Shannon’s information index (*I*) was 0.3230 (Table 4, showed by upright numbers). Parameters of genetic diversity of 29 isolates were calculated by PopGene software and results showed that the mean of *Na, Ne, H* and *I* was 1.9821, 1.7200, 0.4014 and 0.5830, respectively (Table 4, showed by upright numbers). The total heterozygosity (*Ht *= 0.3979), intra-specific heterozygosity (*Hs *= 0.2224), coefficient of gene differentiation (*Gst *= 0.4411) and gene flow (*Nm *= 0.6335) were also calculated for the 29 *L. theobromae* isolates (Table 5, showed by upright numbers).

**Table 3.** Nei’s genetic identity and genetic distance of five populations based on RAPD and ISSR.

Population	GX	GY	GL	GH	GS
GX	–	0.7324	0.6546	0.7632	0.6765
	–	*0.7298*	*0.7533*	*0.8228*	*0.7987*
GY	0.3114	–	0.7091	0.7867	0.6810
	*0.3150*	–	*0.7475*	*0.8061*	*0.8233*
GL	0.4237	0.3438	–	0.7183	0.6984
	*0.2833*	*0.2910*	–	*0.8052*	*0.8128*
GH	0.2702	0.2399	0.3308	–	0.7943
	*0.1950*	*0.2156*	*0.2167*	–	*0.8153*
GS	0.3908	0.3841	0.3589	0.2303	–
	*0.2248*	*0.1944*	*0.2072*	*0.2041*	

Genetic distance (below diagonal) and Nei’s genetic identity (above diagonal), upright numbers for RAPD and italic numbers for ISSR.

**Table 4.** Genetic diversity of five populations based on RAPD and ISSR.

Population	*n*	*Pl*	*Ppl*	*Na*	*Ne*	*H*	*I*
GX	5	89	52.98	1.5298	1.3482	0.1977	0.2924
		*23*	*17.04*	*1.1704*	*1.1520*	*0.0799*	*0.1126*
GY	4	82	48.81	1.4881	1.3747	0.2708	0.2999
		*29*	*21.48*	*1.2148*	*1.1673*	*0.0911*	*0.1313*
GL	6	89	52.98	1.5298	1.3588	0.2015	0.2961
		*65*	*48.15*	*1.4815*	*1.4116*	*0.2187*	*0.3101*
GH	8	130	77.38	1.7738	1.6455	0.3479	0.4952
		*71*	*52.59*	*1.5259*	*1.4004*	*0.2210*	*0.3192*
GS	6	70	41.67	1.4167	1.2775	0.1569	0.2313
		*54*	*40.00*	*1.4000*	*1.3204*	*0.1724*	*0.2468*
Average		92	54.76	1.5174	1.4009	0.2224	0.3230
		*48.40*	*35.85*	*1.3585*	*1.2903*	*0.1566*	*0.2240*

*n*: Sample size; *Pl*: number of polymorphic loci; *Ppl*: percentage of polymorphic loci; *Na*: observed number of alleles; *Ne*: effective number of alleles; *H*: Nei’s gene diversity index; *I*: Shannon’s information index. Upright numbers for RAPD and italic numbers for ISSR.

**Table 5.** Genetic diversity of 29 isolates based on RAPD and ISSR.

	n	Na	Ne	H	I	Ht	Hs	Gst	Nm
Mean	29	1.9821	1.7200	0.4014	0.5830	0.3979	0.2224	0.4411	0.6335
		*1.8444*	*1.5173*	*0.3032*	*0.4528*	*0.2987*	*0.1566*	*0.4756*	*0.5513*
St. Dev		0.1328	0.2663	0.1140	0.1397	0.0136	0.0100		
		*0.3638*	*0.3425*	*0.1717*	*0.2353*	*0.0297*	*0.0109*		

*n*: Sample size; *Na*: observed number of alleles; *Ne*: effective number of alleles; *H*: Nei’s gene diversity index; *I*: Shannon’s information index; *Ht*: total heterozygosity; *Hs*: intraspecific heterozygosity; *Gst*: coefficient of genetic differentiation; *Nm*: gene flow. Upright numbers for RAPD and italic numbers for ISSR.

Of ISSR molecular markers, Nei’s genetic identity and genetic distance were also different among the five populations (Table 3, showed by italic numbers). The genetic identity ranged from 0.7298 to 0.8233, and the genetic distance ranged from 0.1944 to 0.3150. The maximum genetic identity (0.8233) appeared between the population GS and GY, and the maximum genetic distance (0.3150) appeared between the populations GX and GY. Parameters of genetic diversity of the 5 populations and the 29 isolates were calculated by PopGene software and showed that the average value of observed alleles (*Na*) of the 5 populations was 1.3585, the average value of effective alleles (*Ne*) was 1.2903, the average gene diversity index (*H*) was 0.1566 and the average Shannon’s information index (*I*) was 0.2240 (Table 4, showed by italic numbers). The mean of *Na, Ne, H* and *I* of 29 isolates was 1.8444, 1.5173, 0.3032 and 0.4528, respectively (Table 4, showed by italic numbers). The total heterozygosity (*Ht *= 0.2987), intra-specific heterozygosity (*Hs *= 0.1566), coefficient of gene differentiation (*Gst *= 0.4756) and gene flow (*Nm *= 0.5513) were also calculated for the 29 *L. theobromae* isolates (Table 5, showed by italic numbers).

According to Nei’s gene diversity and Shannon’s information index based on RAPD and ISSR molecular markers, the genetic differentiation among isolates was relatively higher, whereas smaller among populations, and there was a high level of genetic diversity among those isolates.

## Discussion

Zhang et al. (2000) found that 30 isolates belonging to the *Botrysphaeria* collected from Shangxi, Liaoning, Hunan, Beijing, Shangdong and Henan province of China had highly polymorphism (92.81%), some isolates showed obvious geographical and host differentiation, and the others did not, according to the UPGMA dendrogram based on RAPD. Our results were partly similar to it, that is, most isolates showed obvious geographical and host differentiation. Sangeetha et al. (2011) reported that 12 isolates of *L. theobromae* collected from 12 different commercial banana cultivars showed high degree of genetic variation and rich genetic diversity among isolates from different banana varieties based on RAPD. Nghia et al. (2012) described that the genetic diversity of 20 isolates of *Botryodiplodia theobromae* (the synonym of *L. theobromae*) and 214 DNA bands were generated with 16 ISSR primers (76.6% of those bands were polymorphic); the clusters of dendrogram showed the relationship among isolates from different regions and no relationship between genetic groups and the hosts. But, the dendrogram generated based on ISSR in our study showed significant correlation between the clusters and both the geographical locations and host plants, and only two isolates collected from Heng County mixed with five isolates collected from Xiangzhou County, the reason of which was probably that mulberry seedlings from Xiangzhou County were transported to Heng County.

The gene differentiation coefficient (*Gst*) denoted the level of genetic variation among populations, and the genetic differentiation among populations was generally large when *Gst* value was above 0.15 (Wright 1978). The strength of gene flow (*Nm*) between populations was the important factors affecting of the population genetic differentiation, and if *Nm* value was more than 1, the gene flow could prevent the differentiation of population that caused by genetic drift, but limited gene flow could promote the genetic differentiation among populations when the *Nm* value was less than 1 (Hartl and Clark 1997). Genetic diversity and gene flow of endophyte *L. theobromae* were studied, and results showed that there was no evidence of host specificity for isolates of *L. theobromae* and there was very high gene flow between populations from different hosts and gene flow was less restricted between isolates from the population of Mexico (Mohali et al. 2005). In this study, the *Gst* value (0.4411 and 0.4756) and *Nm* value (0.6335 and 0.5513) generated from RAPD and ISSR revealed that genetic differentiation was large among isolates and the frequency of gene exchanges between populations was low.

The sample size of *L. theobromae* isolates was relatively small. More isolates collected from different countries, geographical regions, seasons, hosts, ecological surroundings and soil conditions should be studied for thoroughly revealing the genetic diversity and genetic differentiation of *L. theobromae*.

## Highlights

- Mulberry root rot caused by *Lasiodiplodia theobromae* was a new disease in China.
- Genetic diversity of 29 *L. theobromae* isolates was studied.
- Genetic diversity of *L. theobromae* was related to the geographic locations.
